# A Guide to the Clinical Management of *Vipera* Snakebite in Italy

**DOI:** 10.3390/toxins16060255

**Published:** 2024-05-31

**Authors:** Matteo Riccardo Di Nicola, Marta Crevani, Ignazio Avella, Anna Cerullo, Jean-Lou C. M. Dorne, Giovanni Paolino, Caterina Zattera

**Affiliations:** 1 Unit of Dermatology and Cosmetology, Istituto di Ricovero e Cura a Carattere Scientifico San Raffaele Hospital, Via Olgettina 60, 20132 Milan, Italy; 2 Department of Pathobiology, Pharmacology and Zoological Medicine, Faculty of Veterinary Medicine, Wildlife Health Ghent, Ghent University, 9820 Merelbeke, Belgium; 3 Asociación Herpetológica Española, Apartado de correos 191, 28911 Leganés, Spain; 4 Poison Control Centre, Azienda Socio-Sanitaria Territoriale Grande Ospedale Metropolitano Niguarda, 20162 Milan, Italy; 5 Landes-Offensive zur Entwicklung Wissenschaftlich-ökonomischer Exzellenz-Centre for Translational Biodiversity Genomics, Senckenberganlage 25, 60325 Frankfurt Am Main, Germany; 6 Institute for Insect Biotechnology, Justus-Liebig University Giessen, Heinrich-Buff-Ring 26-32, 35392 Giessen, Germany; 7 Department of Veterinary Sciences, University of Turin, L.go Braccini 2, 10095 Grugliasco, Italy; 8 Methodological and Scientific Support Unit, European Food Safety Authority, Via Carlo Magno 1A, 43100 Parma, Italy; 9 Unit of Emergency Medicine, Istituto di Ricovero e Cura a Carattere Scientifico Policlinico San Matteo University Hospital, University of Pavia, P.Le Golgi, 19, 27100 Pavia, Italy

**Keywords:** snakebite, antivenom, snake venom, Viperidae, OldWorld vipers, *Vipera*, *Vipera ammodytes*, Vipera aspis, *Vipera berus*, *Vipera ursinii*

## Abstract

The genus *Vipera* encompasses most species of medically significant venomous snakes of Europe, with Italy harbouring four of them. Envenomation by European vipers can result in severe consequences, but underreporting and the absence of standardised clinical protocols hinder effective snakebite management. This study provides an updated, detailed set of guidelines for the management and treatment of *Vipera* snakebite tailored for Italian clinicians. It includes taxonomic keys for snake identification, insights into viper venom composition, and recommendations for clinical management. Emphasis is placed on quick and reliable identification of medically relevant snake species, along with appropriate first aid measures. Criteria for antivenom administration are outlined, as well as indications on managing potential side effects. While the protocol is specific to Italy, its methodology can potentially be adapted for other European countries, depending on local resources. The promotion of comprehensive data collection and collaboration among Poison Control Centres is advocated to optimise envenomation management protocols and improve the reporting of epidemiological data concerning snakebite at the country level.

## 1. Introduction

Officially recognised as a Neglected Tropical Disease by the World Health Organization [1], snakebites pose a significant public health challenge, with estimates indicating up to 2.7 million people envenomed and around 138,000 fatalities annually, along with approximately 400,000 individuals enduring long-term disabilities [2,3,4,5,6]. Furthermore, these numbers are most likely underestimated, given that the majority of medically significant snakebite accidents occur in some of the most isolated and underdeveloped tropical areas of the world (i.e., Africa, Asia, Latin America, and Papua New Guinea [7,8,9]). In these contexts, challenges in collecting data from remote areas with limited accessibility, unreliable reporting systems, and restricted access to affordable medical care result in inadequate records on the prevalence and distribution of snakebites [3,4,5,6,10].

Snakebites are an often-overlooked issue in Europe [11,12,13,14]. In fact, although Europe is home to a considerable number of medically important snake species, exclusively belonging to the subfamily Viperinae (i.e., “true vipers”, family Viperidae [13,14,15]), people in this continent are rarely exposed to inadvertent interactions with wild snakes, and can usually rapidly access healthcare institutes in case of complications due to envenomation. Nonetheless, despite the reduced annual number of snakebite records, serious and even life-threatening snakebite accidents do occur [11,14]. The relatively low amount of data concerning snakebite in Europe likely contributes to a lack of sufficient interest in the issue, also indicated by the absence of mandatory reporting and standardisation of notification by health services [11,12,13], despite the generally high quality of public health in most parts of the continent. Of all European countries, France and Sweden appear to have a more favorable situation: here, routinely recording human–snake accidents has resulted in the production of structured data on snakebite and improved local clinical management [11].

Italy does not deviate from the overall European trend, lacking mandatory reporting of snakebite accidents or standardisation of notification by health services [16]. Consequently, obtaining up-to-date statistics on snakebites at the national level in this country is not straightforward, and reliance must be placed on scientific literature, often not up to date. Indeed, the most recent analysis of snakebite cases in Italy is reported in a study from almost ten years ago [17], concerning the experience of the National Toxicology Information Centre in Pavia, covering the years from 2002 to 2012. In this time frame, 482 patients were evaluated for viper snakebites. Out of the 482 patients, 170 (35%) did not exhibit signs of poisoning, while 312 (65%) presented symptoms (Grading-Severity-Score [GSS] > 0—see Audebert et al. [18]). Among these, 169 (54% of 312; 35% of the total) manifested severe symptoms (GSS > 1). Antivenom was administered to 44% of patients with clinical signs of poisoning, resulting in clinical improvement in 76% of them. 

For another comprehensive national report, one must go even further back to 1988. In this case, Pozio [19] presented the outcomes of venomous snakebites in Italy, which involved 292 national hospitals between 1980 and 1984. Out of 2329 patients, 1507 (62%) did not exhibit symptoms of envenomation, while 885 (38%) did, with categorisation as minor envenoming for 45% of them, mild for 30%, moderate for 14%, severe for 8%, and fatal for 1% (there were three cases of death).

The clinical management of envenomation following snakebite involves a series of procedures that can vary based on factors such as the snake species involved, the local healthcare system, and the patient’s conditions and observed symptoms [10,20]. In fact, the existing general guidelines for snakebite treatment (see [21,22,23,24,25]) often need to be refined and modified by utilising case-specific protocols. A clinical protocol applicable for first aid and treatment of bites by European vipers of major clinical relevance has recently been published [14]. Although this protocol is applicable at the European level, considering the presence of differences in local healthcare systems and the current availability of treatments and medications among countries, the development of specific protocols for each nation would be desirable. To date, Italy lacks a country-specific clinical protocol for managing snakebites. The objective of this work is to provide a resource that Italian national healthcare personnel can utilise to address symptoms of envenomation resulting from bites of the four *Vipera* species found within the country’s borders, by offering specific information on procedures, communication with poison control centres, and the drugs (including antivenoms) available in Italy.

### 1.1. Italian Vipers: Venom Components and Activities 

Snake venom research has predominantly focused on the family Viperidae, with a particular emphasis on the Crotalinae subfamily and, to a lesser extent, the Viperinae subfamily [26]. Nonetheless, several Viperinae venoms have been thoroughly studied, and a recent comprehensive compilation by Damn et al. [27] has identified four major toxin families constituting about 75% of their compositions. These toxin families include snake venom metalloproteinases (svMP), phospholipases A_2_ (PLA_2_), snake venom serine proteases (svSP), and C-type lectins and C-type lectin-related proteins (CTL). Less abundant toxin families are disintegrins (DI), cysteine-rich secretory proteins (CRISP), vascular endothelial growth factors (VEGF), Kunitz-type inhibitors (KUN), and L-amino-acid oxidases (LAAO). Additionally, minor components such as nerve growth factors (NGF), 5′-nucleotidases (5N), phosphodiesterases (PDE), hyaluronidases (HYAL), phospholipases B (PLB), cystatins (CYS), glutaminyl cyclotransferases (QC), aminopeptidases (AP), SVMP-inhibitors (SVMP-i), and bradykinin-potentiating peptides (BPP) have been observed at low abundances in reported venom compositions.

Among the subfamily Viperinae, the genus *Vipera* is the most frequently involved one in snakebite accidents in Europe [11,13,28]. Specifically, from a recent analysis of 3574 snakebite cases caused by *Vipera* species in the continent, *Vipera ammodytes*, *Vipera aspis*, *Vipera berus*, *Vipera latastei*, *Vipera seoanei*, and *Vipera ursinii* resulted as the six European *Vipera* species of highest medical relevance [13]. Clinical symptoms caused by envenoming by members of this genus range from minimal and local (e.g., algesia, swelling) to systemic and potentially life-threatening (e.g., haematological, neurological [11,13]), and are typically in line with the primarily haemorrhagic and cytotoxic effects associated with viper envenomation [10,20]. Notably, despite the medical relevance of these species, they appear to receive limited attention from snake venom research. Particularly, while the venoms of *V. ammodytes* and *V. berus* have been characterised and analysed with different levels of detail, *V. aspis* and *V. ursinii* seemingly received less attention (see [26,27]), and the venoms of *V. latastei* and *V. seoanei* have been characterised for the first time only very recently [29,30].

In light of the recent comprehensive review on the *Vipera* species of major medical relevance in Europe provided by Di Nicola et al. [14], we present herein a concise overview of the venom components and main activities exhibited by the four *Vipera* species present in Italy, namely *V. ammodytes*, *V. aspis*, *V. berus*, and *V. ursinii*. Considering that Speybroeck et al. [31] deemed it premature to accept the species status of the recently described Walser viper, *Vipera walser* (see [32]) due to the potential existence of cyto-nuclear discordance, which has been confirmed by Doniol-Valcroze et al. [33], in this study we regard this taxon as a subspecies of *V. berus*. This classification aligns with the the findings of a recent phylogenomic analysis of the diversity of Palearctic vipers [34], the latest checklist of the Italian herpetological fauna [35] and the updated dichotomous key for European snakes [36]. Furthermore, it should be noted that there is still a complete lack of studies on the venom of the Walser viper.

#### 1.1.1. Nose-Horned Viper—*Vipera ammodytes* (Linnaeus 1758)

Due to its capacity to induce life-threatening envenomations and its wide distribution [37], the nose-horned viper is listed among the medically significant snake species by the World Health Organization [38], and is traditionally considered the most dangerous venomous snake in Europe [39]. Symptoms caused by *V. ammodytes* venom include haemorrhage and tissue damage, as well as neurotoxicity [40,41,42]. Studies aimed at characterising the protein compositions of the venoms of different subspecies of *V. ammodytes* (*V. a. ammodytes*, *V. a. meridionalis*, *V. a. montandoni*, *V. a. transcaucasiana*) found them to primarily comprise phospholipases A_2_ (PLA_2_), vascular endothelial growth factors (VEGF), snake venom serine proteases (svSP), snake venom metalloproteinases (svMP), L-amino-acid oxidases (LAAO), cysteine-rich secretory proteins (CRISP), and C-type lectins (CTL). Intriguingly, the neurotoxic PLA_2_ vipoxin was the predominant PLA_2_ across the four abovementioned subspecies [43,44]. Recent works, some of which apply both proteomic and transcriptomic techniques, have confirmed this general compositional trend [45,46]. Nonetheless, despite these compositional similarities, LD_50_ experiments have shown that the venom of *V. a. meridionalis* presents higher lethality to lab mice than *V. a. ammodytes* venom, possibly due to the presence of a monomeric phospholipase A_2_ in the former [47].

#### 1.1.2. Asp Viper—*Vipera aspis* (Linnaeus 1758)

Old studies on the venom from the asp viper revealed the presence of proteolytic enzymes, LAAOs, phospholipases, hyaluronidases, hypotensive agents, haemorrhagic agents, and coagulation inhibitors [48,49,50]. Through the recent application of both transcriptomics and proteomics to the analysis of the venom of this species, Giribaldi et al. [51] detected significant amounts of haemotoxins (e.g., svMP of class P-III, CTL, DI) and PLA_2_, which align with the mainly haemotoxic and cytotoxic features of *V. aspis* envenomation [14,52]. By conducing LD_50_ measurements in mice for the two subspecies *V. a. aspis* and *V. a. zinnikeri*, Komori et al. [53] determined that the latter possessed higher lethality. The authors proposed that this disparity could be attributed to the presence of the highly lethal PLA_2_-I in *V. a. zinnikeri* venom, which was absent in *V. a. aspis* venom. Remarkably, neurotoxic effects induced by *V. aspis* have been documented in regions of southern France and Italy [52,54,55], and are thought to be caused by neurotoxic PLA_2_s, particularly ammodytoxin B (also found in *V. ammodytes* venom) and vaspin [28,54,56]. Interestingly, varying levels of PLA_2_ neurotoxins have been detected in different *V. aspis* specimens, possibly influenced by different environmental and physiological conditions [54].

#### 1.1.3. Common Adder—*Vipera berus* (Linnaeus 1758)

The common adder is the most extensively distributed viper throughout Europe, ranging from the United Kingdom to East Asia [57,58,59]. As a consequence of its huge distribution, *Vipera berus* is the species of the genus *Vipera* causing the highest number of snakebite accidents [11,60]. The analysis of venoms from specimens of Russian origin found that serine protease (svSP) and metalloproteinase (svMP) were the dominant venom protein families [61]. Interestingly, a recent work also delving into the venoms of *V. berus* specimens from Russia confirmed the presence of considerable amounts of svSP and svMP, but highlighted PLA_2_s as their most abundant components [62]. Curiously, PLA_2_s emerged as the most abundant proteins of common adder venoms from the Slovakian Republic, comprising nearly 60% of all identified venom proteins, whereas svMP stood as the least prevalent protein group [63]. While these disparities suggest the presence of regional variation in the species’ venom, the compositions reported in these studies are consistent with the primarily cytotoxic, haemolytic, and proteolytic properties of *V. berus* venom [28,64,65]. Nonetheless, it should be noted that Malina et al. [66] documented predominantly neurotoxic neuromuscular activity in *V. berus* venoms from Hungary, as well as differences in protease and phospholipase activity among specimens of different sexes and ages. These results further support the presence of venom variation within this species. 

#### 1.1.4. Meadow Viper—*Vipera ursinii* (Bonaparte 1835)

The meadow viper is often regarded as the least medically relevant member of the genus *Vipera*. This is mainly due to the limited amount of venom it can inject, and the mild and localised symptoms that envenomations caused by this species typically elicit in humans [67,68]. The venom of *Vipera ursinii* has been documented to cause haemorrhagic effects in mice [69]. Consistent with this, a recent study found that svMPs of class P-III are the most abundant components of *V. ursinii* venom, accounting for more than half of all identified venom proteins [70]. Interestingly, proteomic analyses performed on the venom of the closely related *Vipera anatolica* provided similar results [71,72]. The haemorrhagic effect of *V. ursinii* venom is likely exacerbated by other proteins detected in it, such as svSPs, known to induce coagulopathy and affect haemostasis [73]. Notably, the injection of meadow viper venom has been found to elicit symptoms suggesting neurotoxicity in both mice and crickets, and to be more toxic to crickets than mice [70]. The latter finding indicates that *V. ursinii* venom likely possesses marked specificity for orthopterans, accounting for the majority of this species’ diet [67,74].

## 2. Results—Clinical Management of *Vipera* Envenomation

### 2.1. Identifying the Biting Snake

Knowing which snake species inhabit a region and understanding how to distinguish them is useful both to avoid unnecessary concern in the event of a bite by a non-medically relevant species, and to obtain the most precise information in case of actual envenomation. Regarding the latter aspect, having information about the species responsible for the envenomation can facilitate the specificity and promptness of treatments (e.g., the choice of antivenom). Taking into account also allochthonous taxa, Italy currently hosts 22 species of snakes belonging to six different families (considering subspecies as well, the taxa are 27; see Table S1 and the figures cited therein). Of these species, only four, all belonging to the genus *Vipera*, are officially categorised as medically important by the World Health Organization [75]: *Vipera ammodytes*, *Vipera aspis*, *Vipera berus*, and *Vipera ursinii*. Two non-front-fanged colubroid (NFFC) snakes of the family Psammophiidae, *Malpolon monspessulanus* and *Malpolon insignitus*, can also be found in Italy (with a national distribution limited to the central-western Liguria and the island of Lampedusa, respectively—see [76,77]). These two species are generally believed to induce only mild, local envenomation symptoms, although some reports indicating more severe symptoms exist [15,78,79,80,81]. Two additional species of NFFC venomous snakes, namely *Telescopus fallax* and *Macroprotodon cucullatus*, can also be found in Italy, but there are currently no official reports of envenomation caused by them in humans [82]. As none of these four NFFC species are deemed medically important by the World Health Organization [75], they will not be considered in the present study.

#### 2.1.1. Distinguishing Vipers from other Italian Snakes

Observing the morphological characteristics reported in the following identification key allows for a reliable distinction between vipers, the only reptiles of medical importance in Italy, and other snake species found in the country (Table S1). The provided indications are based on the dichotomous keys by Di Nicola [76] and Di Nicola et al. [36]. For the counting and nomenclature of the main scales used for species identification, refer to Figures S5, S6 and S7.

**A1**. Snake-like appearance. Eyes well developed and visible. Ventral scales larger than dorsal ones………………………………………………………………………………….**B**

**A2**. Worm-like appearance and very small size (up to 17 cm in total length). Dorsal and ventral scales very similar to each other; spiny scale on the tip of the tail. Snout rounded in profile, very small eyes protected by a semi-transparent shield and visible only as a dark spot. Allochthonous species, currently found only in the Province of Trapani (Sicily) and on Ischia Island (Campania)…................................................................................... ……………………………………………….**Not a viper** (*Indotyphlops braminus*, Figure S1A).

**B1**. Head more or less distinct from the body. Slenderer body, tail longer and pointed. Bigger eyes, with round or vertical pupil. Subcaudal scales paired. Ventral scales almost entirely covering the width of the belly. Less than 30 rows of dorsal scales counted at mid-body.................................................................................................................................................**C**

**B2**. Head not distinct from the body. Bulky body, tail short and blunt. Small eyes, with vertical pupil. Subcaudal scales (mostly) in a single row. Ventral scales wide and covering less than half the width of the belly; remaining belly surface covered with small and smooth identical scales as for the dorsum. Dorsal scales of the front half of the body smooth, keeled towards the end. Forty-one to fifty-seven rows of dorsal scales counted at mid-body. Possibly introduced in historical times; found only in a limited coastal area of the provinces of Agrigento and Caltanissetta (Sicily)…………..………**Not a viper** (*Eryx jaculus*, Figure S1B).

**C1**. Top of the head covered by 9–11 large, smooth and symmetrical shields. Anal plate usually divided. Eyes with round pupil in combination with smooth, keeled or grooved dorsal scales, or eyes with vertical pupil only if in combination with smooth dorsal scales. Supralabial scales touching the eye (except for a species distributed only in the Province of Cagliari, Sardinia, and on Pantelleria Island, Sicily, due to the presence of small subocular scales). Species present in all regions of mainland Italy, Sardinia, Sicily, and many smaller islands…………………………………………………………………… ….**Not a viper** (Colubridae, Natricidae and Psammophiidae species, Figures S2A–S4D).

**Figure 1:**
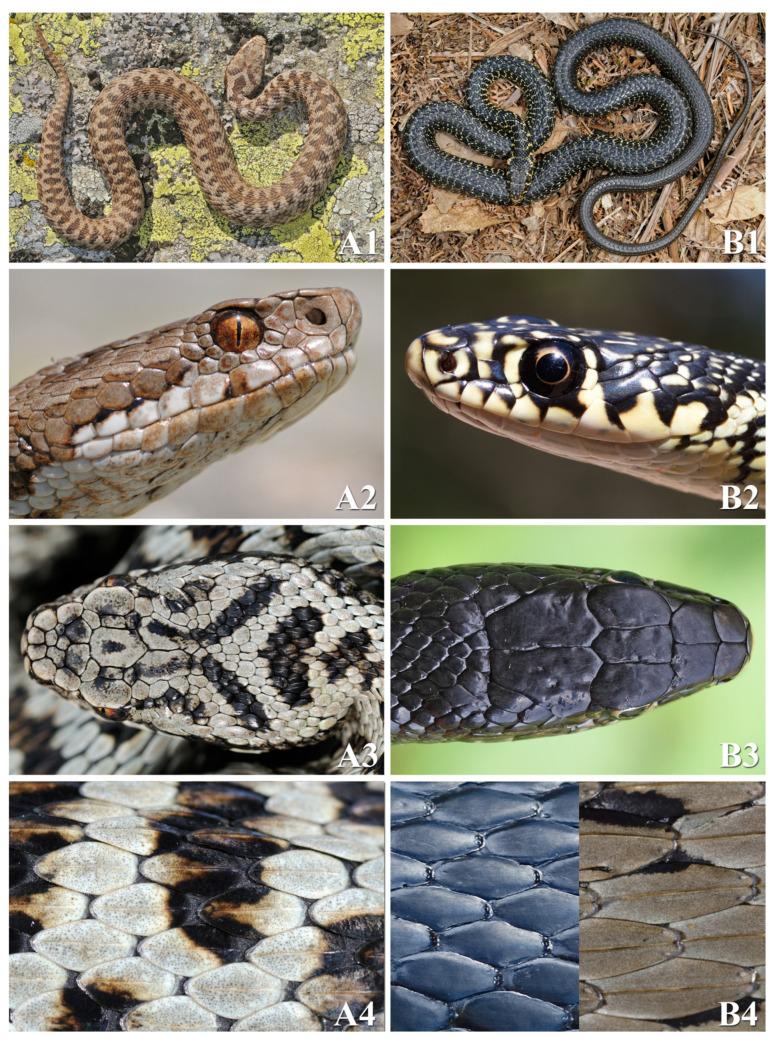
Main morphological differences between European vipers (**A**) and colubrids *sensu lato* (**B**). The body of vipers (**A1**) is proportionally shorter and stockier than that of colubrids (**B1**), with a lower length-to-width ratio. Additionally, Italian vipers usually reach an average size of 50–70 cm and do not exceed one metre. Various species of Italian colubrids, on the other hand, frequently exceed one metre in length. The pupil of vipers (**A2**) is vertical and in full light takes on a slit-like shape; the pupil of colubrids (**B2**) is round. The only exception is *Telescopus fallax*, a colubrid found only in extreme north-eastern Italy, which has a slit-like pupil. Vipers (**A2**) have at least one row of subocular scales that separate the eye from the supralabial scales; colubrids (**B2**) have the eye in contact with the supralabial scales. The only exception is *Hemorrhois hippocrepis*, a colubrid found in southern Sardinia and Pantelleria. Vipers (**A3**) have the top of the head covered by small scales arranged irregularly or, at most, three shields arranged symmetrically, surrounded by smaller scales; colubrids (**B3**) have the top of the head covered by 9–11 large smooth shields symmetrically arranged. This characteristic has no exceptions. Vipers (**A4**) have dorsal scales on the trunk that are always keeled; colubrids (**B4**) can have dorsal scales on the trunk that are smooth or keeled depending on the species (in Psammophiidae, dorsal scales are grooved). Finally, the shape of the head is not a reliable distinguishing characteristic: vipers can have a more or less triangular or a sub-oval head shape; some colubrids can also have (or adopt, as a defensive posture) a more or less triangular head shape. The species portrayed in the pictures are *Vipera berus* (**A1**–**A4**), *Hierophis viridiflavus* ((**B1**–**B4**) L), *Natrix helvetica* ((**B4**) R). Photo credits: Matteo R. Di Nicola.

**Figure 2:**
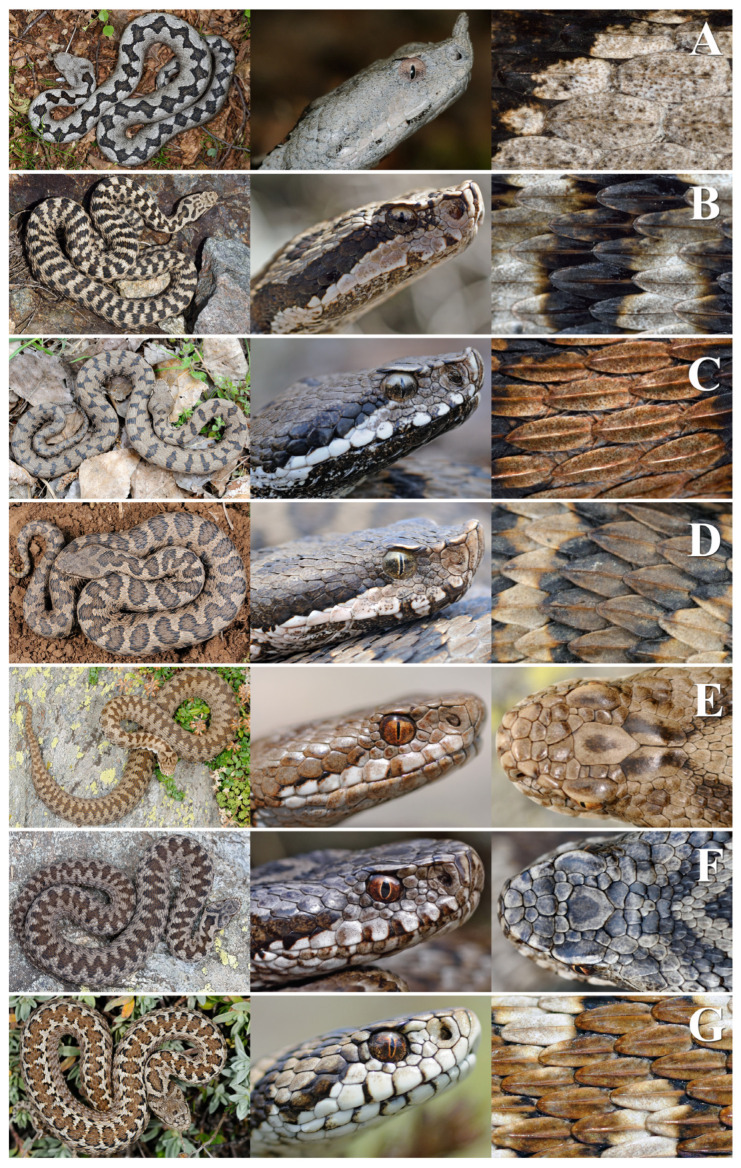
Photos of the species and subspecies of Italian vipers: *Vipera ammodytes ammodytes* (**A**), *V. aspis aspis* (**B**), *V. a. francisciredi* (**C**), *V. a. hugyi* (**D**), *V. berus marasso* (**E**), *V. b. walser* (**F**), *V. ursinii ursinii* (**G**). For each taxon, examples of dorsal pattern, head portrait, and dorsal scales are shown. For *V. b. marasso* and *V. b. walser*, cephalic scales rather than dorsal scales are shown for comparison. Image from Di Nicola (2019) [76].

**C2**. Top of the head covered by small scales irregularly arranged, or at most three shields symmetrically arranged, surrounded by smaller scales. Anal plate usually entire. Eyes with vertical pupil (slit-like in daylight) always in combination with keeled dorsal scales. No supralabial scales touching the eye due to the presence of small subocular scales. Species present in all regions of mainland Italy, Sicily, Elba, and Montecristo islands; absent from Sardinia and the remaining smaller islands…………………………….…………………………………………….............................***Vipera* spp.**, Figure 1(A1–A4) and Figure 2A–G. 

Figure 1 summarises the main morphological features for quickly distinguishing Italian vipers from colubrids *sensu lato* (i.e., Colubridae, Natricidae, and Psammophiidae).

#### 2.1.2. Which Viper Is It?

**A1**. Tip of the snout dorsally flat; frontal and/or parietal scales usually larger than surrounding scales; one or two rows of suboculars………………………………………..….**B**

**A2**. Tip of the snout more or less upturned or with a scaly horn; frontal and parietal scales most of the time replaced by small scales; two rows of subocular scales (occasionally one, rarely three)……………………………………………………………………………………….. **D**

**B1**. A total of 19 rows of dorsal scales at mid-body (occasionally 21); 1 apical scale (rarely 2); superior preocular scale usually in contact with the nasal one; 7–9 supralabial scales; 1 row of subocular scales; 120–130 ventral scales; 20–32 pairs of subcaudal scales; adult total length usually below 50 cm; species present only in the central Apennines of Abruzzo, Marche, Lazio, and Umbria…………………………………………… ………………………………………………………………..…..***Vipera ursinii ursinii***, Figure 2G.

**B2**. A total of 21 rows of dorsal scales at mid-body (occasionally 19–23); two apical scales (rarely one); superior preocular scale usually not in contact with the nasal one; nine supralabial scales (occasionally 8); adult total length commonly above 50 cm; species present only in the Alps……………………………………………………………………….…………..…..…..**C**

**C1**. A total of 131–165 ventral scales; 22–43 pairs of subcaudal scales; usually 1 row of subocular scales; an averagely lower count of cephalic scales compared to the next taxon: 4–22 crown scales, 2–10 parietal scales, 2–12 loreal scales of both sides, 13–23 periocular scales; taxon present only in the Alps in Lombardy, Trentino-Alto Adige, Veneto, and Friuli-Venezia Giulia.…..……………………………………...***Vipera berus marasso***, Figure 2E.

**C2**. A total of 138–156 ventral scales; 23–38 pairs of subcaudal scales; 1,5–2 rows of subocular scales (occasionally 1); a greater tendency to fragmentation of cephalic scales, resulting in an averagely higher count: 7–30 crown scales, 2–14 parietal scales, 4–15 loreal scales of both sides, 16–23 periocular scales, frontal scale occasionally fragmented into smaller ones; taxon present only in the Alps in north-eastern Piedmont. Appearance very similar to the previous taxon, with a considerable overlap of pholidotic values. Without genetic characterisation, identification certainty is only possible based on the area of discovery...…….……………………………………………………...***Vipera berus walser***, Figure 2F.

**D1**. Tip of the snout more or less upturned because of high rostral scale; 2–4 small scales covering the raised part of the snout; 21 rows of dorsal scales at mid-body (occasionally 22–23, rarely 17–24); 9–11 supralabial scales. Presence of three subspecies with hybridisation areas and intermediate phenotypes occurring at the boundaries of their distributions (*Vipera aspis* spp.)…………………………………………………………………….**E**

**D2**. Tip of the snout with horn covered by 5–20 small scales (rostral scale not extended to the horn front); 21 rows of dorsal scales at mid-body (occasionally 20–23); 9–10 supralabial scales (rarely 8–12); 128–169 ventral scales; 22–44 pairs of subcaudal scales. Species present only in Friuli-Venezia Giulia, South Tyrol, and Veneto………………………………………………...***Vipera ammodytes ammodytes***, Figure 2A.

**E1**. Averagely 154 (MM)–155 (FF) ventral scales (range: 148–161); anterior labials usually not visible from above; dorsal pattern with segments separated or partially in contact to form a zig-zag; subspecies present in Aosta Valley, most of Piedmont, central and western Liguria, and south-western Lombardy………………….....***Vipera aspis aspis***, Figure 2B.

**E2**. Averagely fewer ventral scales; subspecies present in the rest of Italy (excluding Sardinia)…….………………………………………………………………..…..…………..**F**

**F1**. Averagely 146 (males, MM)—150 (females, FF) ventral scales (range: 136–159); anterior labial scales usually visible from above in adults because slightly protruding; dorsal pattern with segments usually separated and more spaced, resulting in a smaller number. Subspecies present in some areas of eastern Piedmont, most of Lombardy, Veneto, Trentino-Alto Adige, Friuli-Venezia Giulia, Emilia-Romagna, Tuscany, Marche, Umbria, Lazio, Abruzzo, Molise, northern and central Campania, northern Basilicata, northern Apulia, and on Elba Island……………………….….….***Vipera aspis francisciredi***, Figure 2C.

**F2**. Averagely 141 (MM)–145 (FF) ventral scales (range: 135–150); anterior labial scales usually not visible from above; dorsal pattern with elliptical, roundish or quadrangular shapes arranged in a zig-zag; subspecies present in central and southern Campania, Apulia, and Basilicata (excluding the northernmost portions), Calabria, Sicily, and (introduced) on Montecristo Island………..…………………………...***Vipera aspis hugyi***, Figure 2D. 

### 2.2. First Aid in the Field

Following a suspected viper bite, the primary concern for the patient is to maintain composure and promptly call an emergency number to secure admission to an Emergency Department (ED). Anxiety and physical agitation can exacerbate venom dispersion by increasing blood flow. It is crucial to understand that minimal intervention before reaching a hospital setting generally yields optimal outcomes for the patient. Despite common misconceptions, practices like applying compression, using tourniquets, making incisions, attempting oral suction, applying ice, employing heat or chemical agents, or administering electric shocks locally are not supported by scientific evidence and may in fact increase the risk of clinical complications (see [10,83,84,85,86]). 

In outdoor environments, the most beneficial actions include cleansing the wound with water and alcohol-free detergents, removing any constricting objects (e.g., jewellery, watches) that might impede blood circulation in case of swelling, and applying an immobilisation bandage. However, due to the challenges associated with correctly applying such bandages to avoid excessive compression, their application should be entrusted solely to emergency personnel and promptly removed upon hospital admission. The primary focus for emergency services is therefore to prioritise the speed of patient transportation, utilising cars or helicopters for the most remote areas. 

Nonsteroidal Anti-Inflammatory Drugs (NSAIDs) should be avoided due to their potential to increase bleeding risk. Instead, paracetamol can effectively manage pain and aid in controlling stress and agitation. When warranted and in the absence of clinical contraindications or signs of neurotoxicity (see [87]), low-dose benzodiazepines and/or opioids may be administered to alleviate anxiety symptoms. If feasible and without heightening the risk of further bites, taking a photograph of the snake for later identification by an expert herpetologist should be considered.

### 2.3. Laboratory and Clinical Investigations

Once the patient is admitted to the hospital, a Poison Control Centre (PCC) should be consulted as soon as possible to help the clinician in the evaluation, treatment, and follow-up of the patient. PCCs are emergency services available 24/7, providing specialised telephonic consultation to hospitals and the general public regarding the diagnosis, treatment, and prevention of acute intoxications (Table 1).

First, it must be confirmed if an envenomation occurred. If venom has been injected, local symptoms can arise in a matter of minutes, while systemic symptoms generally take longer to appear [20]. The grading of the envenomation must be assessed [88], and the patient needs to be observed and monitored for some hours after the bite. If early signs of envenomation are not detected, 6–8 h of observation are sufficient. Usually, local symptoms develop within two hours after the bite, although some systemic conditions may take longer to appear, but always in association with local manifestations [55]. Not involving the injection of any venom, dry bites are generally much less painful than venomous ones, but fright can often represent a bias, emphasise the pain and, in extreme cases, even lead to death (see [89]). As a general rule, baseline laboratory investigations should be always performed for each patient, including glycaemia, blood count, renal and hepatic function, coagulation tests, rhabdomyolysis indexes, and an electrocardiogram (ECG). All these investigations should be reassessed according to the evolution of the clinical picture and others may be conducted based also on the patient’s clinical history. If no clinical symptoms of envenomation and no laboratory alterations occur during the observation period after the bite, the patient is safe for discharge from medical care, with instructions to return if new pain or swelling develops. Notably, a computerised tool to help clinicians in a rapid evaluation of *Vipera* envenomation has recently been developed (i.e., VipGrade^®^; [90]), and represents a potentially valuable supplement to the advice given by the toxicologists of the PCCs. 

### 2.4. Supportive Treatment of Local Symptoms 

In most cases, the bitten area is characterised by the presence of two fang marks spaced approximately 6 mm to 8 mm apart, depending on the species and size of the biting snake. Sometimes only one fang mark can be seen, which can make it difficult to distinguish between a viper bite or a bite from a different snake. A blood drip can be present in the puncture sites, and the surrounding area can become swollen and painful soon after the bite [91]. Concerning bites to the hands or feet, an initial swelling around the puncture wounds can rapidly extend to encompass the entire limb within a matter of hours. Ecchymosis accompanied by blisters can be present, and loco-regional lympho-adenopathy may be indicative of the spread of the venom into the lymphatic system. Haemorrhagic vesicles and blisters may eventually arise in the bitten area within 12 h after the bite and their extension can indicate an underlying necrosis (5.5% of envenomation cases due to European *Vipera* bites [40]). It must be pointed out that even patients experiencing “dry bites” may develop symptoms, likely related to anxiety and vagal reactions, which can be mistakenly attributed to early signs of envenoming by the clinician. The challenge of correctly identifying a venomous bite is further complicated by the fact that local symptoms can appear before, simultaneously, or even after systemic ones. Indeed, although local symptoms generally appear before systemic ones, the sequence of events related to the venom spread can vary. Therefore, the Grading Severity Score (GSS) is assessed based on the evaluation of both the clinical picture (i.e., local and systemic symptoms) and the laboratory findings. 

As soon as the patient is admitted to the hospital, local disinfection with hydrogen peroxide should be performed; alcohol and other chemical substances should be avoided since toxic compounds can be generated [23]. The leading edge of the swollen area must be marked with a dermographic pen and monitored hourly, taking serial circumferential measurements of the involved limb at multiple points proximal to the wound to identify the progression of edema and the appearance/evolution of any other local symptom. Only in case of the development of important local oedema, the affected limb may be slightly raised [92] considering that the elevation can affect the reliability of the measurements. Although there are no studies evaluating whether limb elevation can influence the outcome of the envenomation, it could decrease dependent edema, reduce pain and the risk of compartment syndrome.

Based on historical data, it is evident that the percentage of ulceration and massive necrosis is notably lower in instances of bites from European vipers compared to bites from other Viperidae species (e.g., [40,91]). In these cases, a swab of the affected area is recommended for diagnostic purposes, and a systemic antibiotic treatment (starting with empiric antibiotic therapy, subject to modification contingent upon the antibiogram findings) together with local wound management should be performed [93]. In rare cases, compartment syndrome may develop. This is defined by an excessive pressure inside an enclosed muscle space in the body, reducing blood flow to the tissues with consequent massive and rapid necrosis [94]. An urgent surgical evaluation and intra-compartmental pressure monitoring is required and, if compartment syndrome is diagnosed, a fasciotomy may be recommended [95,96]. There are some factors that can be predictive of the development of compartment syndrome, especially following bites in the limbs. For instance, children, due to their lower volume of distribution and higher plasma venom concentration, are at higher risk (e.g., [97,98]). Some authors suggest that an elevated white blood cells (WBC) count and increased aspartate aminotransferase (AST) level upon admission to the emergency department (ED) may be useful as clinical markers and risk factors for the development of compartment syndrome [99].

Concerning snake bites inflicted to the fingers, since the digit lacks distinct compartments, swelling resulting in tissue necrosis would not technically be classified as compartment syndrome [100]. Nevertheless, neuro-vascular bundles within the digit serve as small compartments. When the skin of the digit reaches its elastic limit, pressure within the “compartment” may exceed the capillary closing pressure, and the integrity of small vessels and nerves may be compromised. In such cases, studies demonstrate that dermotomy and decompression of the entire finger must be performed as soon as possible [100,101]. Other supportive treatments including mannitol and hyperbaric oxygen therapy have been proposed, but are not commonly used in clinical practice [102,103].

### 2.5. Supportive Treatment of Systemic Symptoms

The initial in-hospital assessment of patients suspected of viper envenomation should always focus on airway, breathing, and circulation. In acute and severe cases of viper bite, patients may develop an anaphylactic shock or an anaphylactoid reaction occurring with circulatory collapse and angioedema a few minutes after the bite. It is important to highlight that such reactions can appear in both previously exposed and unexposed patients [104,105]. In the case of anaphylaxis, rapid primary clinical assessment and resuscitation using the ABCDE (Airway, Breathing, Circulation, Disability, Exposure/Environment) approach are needed, then intramuscular adrenaline should be quickly administered according to the updated guidelines of the European Academy of Allergy and Clinical Immunology [106]. 

It is essential for the patient to stay calm, in order to lower heart rate and thereby reduce the spread of the venom through the body. Hence, when necessary, benzodiazepines can be administered, except in cases manifesting clinical signs or symptoms of neurotoxicity or proven bleeding or coagulopathy, due to reported venom potentiation [87]. Although gastrointestinal symptoms (e.g., nausea, vomiting, diarrhea, abdominal pain) are usually the first systemic signs of envenomation [21,23], they can also be the consequence of fear and vagal reaction. Therefore, these symptoms, especially when mild, are not to be considered a direct indication for the administration of antivenom. Nonetheless, gastrointestinal symptoms should always be evaluated carefully, particularly in children, who have an increased risk of complications because of the potential greater distribution of the venom in relation to their body weight [21]. In 2021, Marano and colleagues proposed a Grading Severity Score adjusted for pediatric patients (pGSS) with the relative management indications, which can prove to be a very useful ready-to-use tool for the emergency pediatricians [107].

The tetanus immunisation status of the patient should always be assessed and updated when appropriate. There is no clinical evidence about the benefit of corticosteroid administration except for treatment of anaphylaxis, since oedema is due to a capillary leak and impaired lymphatic drainage rather than inflammation, and corticosteroids treatment did not prove to be effective in reducing hospital stay. Moreover, corticosteroids increase the risk of bacterial infections. Both Swedish and French clinical experience do not recommend their administration [88,108,109]. Regarding antibiotic treatment, it is generally not advised, and a prompt selected administration should be started only when infection is suspected [88,109].

Neurotoxicity has been proven to be caused by the action of PLA_2_ from the venom of *Vipera ammodytes* and some populations of *Vipera aspis* and *Vipera berus* [28,54,66,110]. Neurotoxic symptoms caused by *Vipera* bites have been reported to consist of cranio-caudal paralysis starting from cranial nerve involvement, and include ptosis, ophthalmoplegia with double or fuzzy vision, dysphonia, dysphagia, dysarthria, a variable degree of facial muscle paralysis, and general muscle weakness [42,54,111,112]. According to Lonati et al. [55] patients presenting only ocular neurotoxic effects associated to mild local effects and/or mild gastrointestinal symptoms do not need antivenom treatment, since neurotoxicity induced by the European viper population does not appear to induce respiratory failure muscle paralysis in the absence of other systemic symptoms. Despite this observation, a rapid worsening of local effects and/or the appearance of severe neurologic manifestation like dyspnea could take place and would indicate the need for antivenom treatment. Since Lonati et al. [55] describe the appearance of neurologic symptoms at least 11 h after the bite even in patients with only mild local swelling, they suggest that patients with moderate to severe local symptoms should be monitored for at least 24 h for the risk of a late appearance of neurotoxicity, which can be the only systemic manifestation. On the contrary, they point out that neurotoxicity in pediatric patients, even if it can be the only systemic manifestation, is always associated with extensive local effects. 

In the past, in order to predict the effectiveness of anticholinesterase (neostigmine) treatment to improve neurologic symptoms by increasing acetylcholine transmission in a post-synaptic blockade of neuro-muscular transmission (like in myasthenia gravis patients with ptosis), the Tensillon test used to be performed. Because of the risk of adverse reactions and falsely positive results, this test is no longer used in clinical practice. The “ice pack test” has been proposed as a possible alternative, but it has not been evaluated in patients with neurotoxic manifestation after *Vipera* bites [23]. To perform this test, in the case of a patient with bilateral ptosis from a neurotoxic snakebite, the distance between the upper and lower lid margins of both eyes (palpebral fissures) is measured in mm using a ruler. Digital pressure is applied to the frontalis muscle to avoid its influencing upper eyelid retraction. An ice-filled plastic glove or frozen ice pack is then gently applied to one eyelid for 2 min, after which the palpebral fissures of both eyes are immediately re-measured. A more-than-2 mm difference in palpebral fissure between the cooled and control eyelids might be considered a positive result [113]. 

Coagulopathy and haemorrhagic effects are common symptoms of viperid envenomation [10,20,114], and they can lead to consumption disorders resembling Disseminated Intravascular Coagulation (DIC). Recently, the term “venom-induced consumption coagulopathy” (VICC) has been introduced to describe this condition, which can, in some cases, be associated with thrombotic microangiopathy (TMA). While VICC has been thoroughly documented in the context of Australian elapids and some viperid snakes like *Daboia russelii*, it remains a rare condition in cases of European viper envenomation. Maduwage and Isbister [115] cite *V. aspis*, *V. berus*, and *V. ammodytes ammodytes* as potentially responsible for this complication. The exact mechanism underlying VICC and TMA is still unclear. Antivenom administration is currently the main treatment for VICC, although there is still limited high-quality evidence to support its effectiveness, particularly in envenomations by Echis spp. While heparin has not been shown to improve VICC outcomes, fresh frozen plasma should be considered for bleeding patients to expedite the recovery of coagulopathy. In cases of TMA manifestations, the American Society of Apheresis considers plasmapheresis a weak recommendation that must be individualised for each clinical case [116]. To date, there are no reports of TMA treated with plasmapheresis in patients bitten by European vipers.

Clinicians should be very careful in the preventive administration of Low Molecular Weight Heparin (LMWH) to bitten patients. Boels and colleagues advise not to administer LMWH preventively, since it can significantly increase the incidence of haematomas. Additionally, patients treated with LMWH have shown an increased length of hospital stay and functional impairment [88]. Other authors suggest the risk of aggravate tissues bleeding when high-dose intravenous heparin is administered [108]. Therefore, it is reasonable to consider the administration of heparin only in the case of confirmed deep venous thrombosis, in the context of compartment syndrome.

All the other systemic manifestations of viper bites must be managed symptomatically according to usual clinical practice. 

### 2.6. Specific Treatment: Antivenom Administration

Although snake envenoming is a rare event in Europe, estimated to affect 0.4–1.1 people per 100,000 population per year, it still is a considerable threat, potentially causing severe complications, long-term sequelae, and (rarely) death [11]. In June 2017, WHO formally listed snakebite envenoming as a highest priority neglected tropical disease, and started to develop a comprehensive strategy aimed at reducing the negative impact of snakebite worldwide. One of the main aims of this strategy was to guarantee access to safe and effective antivenoms, capable of directly neutralising the toxic effects of venom components [6]. Currently, all the antivenoms available on the market are enzyme-digested equine or ovine immunoglobulins of the G class in the form of F(ab’)_2_ and Fab fragments raised against venom from a single (monovalent) or several (polyvalent) snake species. Due to the variable molecular mass of the active compound, they exhibit different pharmacokinetic profiles, as outlined by a comprehensive analysis mainly focusing on antivenoms against the venoms of viperids from the global snakebite hotspots [117]. Typically, F(ab’)_2_ fragments have a higher molecular weight, can persist in the circulation for a longer time (median half-life of 2–4 days) and have two antigen-binding sites that allow the creation of stable, multivalent immune-complexes with toxins, which are cleared by phagocytic cells of the reticuloendothelial system. On the contrary, Fab fragments, thanks to their lower mass, can easily reach extravascular compartments and are eliminated by the kidneys with a median half-life of 2–24 h. That is the reason why in some cases, Fab fragments’ administration may be repeated, considering the redistribution of the venom into the circulation by slow continuous absorption from the bite site. Based on these features, it is important to consider the best antivenom format according to the type of envenoming: Fab antivenoms are considered more suitable for elapid venoms, rich of low-molecular mass toxins, while F(ab’)_2_ antivenoms proved more effective in counteracting larger molecules characteristic of viperids [118].

Ideally, venom–antivenom binding should occur during or even prior to the delivery of venom components from the bite site to the place of action, since once the symptoms are established the antivenom efficacy proportionally diminishes. There is still debate on which route of administration (i.e., intramuscular or intravenous) is the most appropriate. WHO recommends that, whenever possible, snake antivenoms should be given intravenously (IV), where they exert a higher speed of distribution and greater bioavailability compared to other routes [119]. A slow infusion over 30–45 min allows a rapid cessation of the treatment in case of immediate adverse reactions. It must be performed under the supervision of healthcare professionals in a hospital setting. The intra-muscular (IM) administration brings a lower risk of antivenom-associated side effects, but there is still debate on the lower efficacy of this route [117]. The WHO advises the IM route only as an alternative approach for patients who are far from medical facilities or whenever IV access cannot be taken. Since in Italy the National Pharmaceutical Agency (AIFA—Agenzia Italiana del Farmaco) allows the administration of snake venom antisera only in hospital settings and transportation to the hospital is often rapid (even in remote areas of the Country), the IV route is the most used [120].

A recent work comparing efficacy and safety of the antivenoms against European *Vipera* spp., currently used in clinical practice in Europe, provides an accurate description of all the different antisera available on the market [121]. This study lists eight antisera: Viper Venom Antitoxin^®^; Snake Venom Antiserum^®^; Anti-viper Venom Serum^®^; Vetal Polisera^®^; Viekvin^®^; ViperaTab^®^; ViperFAV^®^; European Viper Venom Antiserum^®^ (see details in Table 1 of Lamb et al. [121]). Of these, five are monovalent, raised against the venoms of *Vipera berus* (Viper Venom Antitoxin^®^, ViperaTab^®^) or *V. ammodytes* (Snake Venom Antiserum^®^, Viekvin^®^, European Viper Venom Antiserum^®^). Of the remaining three, two are polyvalent: one (Vetal Polisera^®^) raised against the venoms of *Vipera* ammodytes, *Macrovipera lebetina* and *Montivipera xanthina*, and the other (ViperFAV^®^) raised against the venoms of *Vipera berus*, *V. aspis*, and *V. ammodytes*. For the last one, the Anti-viper Venom Serum^®^, a safety data sheet and information are not available online. Only one of these antivenoms, i.e., ViperaTab^®^, which comprises specific immunoglobulin (Fab) fragments raised against *Vipera berus* venom produced by MicroPharm Limited (UK), received the orphan designation (EU/3/15/1548) from the European Medicines Agency in October 2015 [122]. In their work, Lamb et al. emphasise that IV administration of the antivenoms is generally associated with a shorter hospital stay. This is due not only to the faster onset of action via this route, but also to a reduction in complication and overall hospital stay thanks to the improved purification methods applied to current antivenoms [121]. It has to be noted that the European Viper Venom Antiserum^®^ is no longer available in the Italian market, and the Anti-Viper Venom Serum^®^ cannot be considered due to the unavailability of its datasheet and information online. Furthermore, for Vetal Polisera^®^, there is a lack of data regarding any importation and use in our Country. In Table S2, the six antivenoms currently available in Italy are listed.

Currently, there is no standardised protocol for antivenom administration in Europe; therefore, every country follows different indications. Additionally, only Sweden and France developed their own systems for envenomation grading (see Table 2 ). According to Swedish Stockholm criteria [123], the antivenom should be administered in case of hypotension and circulatory shock, protracted severe gastrointestinal symptoms, mucous membrane oedema with a risk of bronchial obstruction, rapid extension of oedema to an entire limb and/or to the torso, neurological symptomatology with depressed central nervous system (CNS) and peripheral and central paresis. In cases of insufficient clinical signs, the antivenom may be administered when any of the following conditions are fulfilled: leukocytosis exceeding 15–20 × 10^9^/L, elevation of AST (aspartate aminotransferase), ALT (alanine aminotransferase), CPK (creatine phosphokinase) or other enzymes, metabolic acidosis, haemolysis, electrocardiogram (ECG) changes, and coagulation disorders. 

While the Stockholm criteria are less known, the grading proposed by Audebert et al. [124], then modified by Boels et al. [88], is still the most used in clinical practice to guide antivenom administration, and recommends the prescription of antivenom as soon as the envenoming progresses to Grade 2. As previously mentioned, Marano and colleagues [107] revised the GSS for the assessment and treatment of pediatric patients. They incorporated specific cutoff values for white blood cell count, neutrophil percentage, and INR, along with corresponding management guidelines.

However, it is of paramount importance to consider that the indication to administer antivenom must be always discussed first with the local PCC, which can also provide the antidote in case of unavailability.

### 2.7. Envenoming Grading Systems

The amount of venom injected in both adults and children is assumed to be the same; therefore, the dosage of antivenom to be administered is the same, independently from the patient’s age and weight. This is usually one or two ampoules, depending on the indications of both the manufacturer and the toxicologist of the PCC. Some cases require one or more additional administrations of antivenom, typically because of the suboptimal therapeutic efficacy of the initial treatment, or in case of recurring clinical manifestations (often involving coagulopathy).

Since antivenoms are preparations of immunoglobulins purified from plasma of hyperimmunised animals, their administration carries the risk of early and late adverse reactions [125,126]. According to the classification proposed in 2010 and updated in 2016 by the WHO [23], early reactions are those that occur within 24 h after antivenom administration, and they can be pyrogenic reactions (rare) or anaphylactoid reactions (more common). Anaphylactoid reactions can be divided into two categories: (1) IgE-mediated: characterised by type I hypersensitivity according to the old Gell–Coombs classification [127], these can occur in patients who were previously exposed to animal immunoglobulins, and produced IgE towards antivenom proteins; (2) non-IgE-mediated reactions: most early reactions, the pathogenesis of which is still incompletely understood. Since these reactions are not IgE-mediated, intradermal tests do not prove useful to predict their occurrence, and are therefore not recommended [128].

The rate of early adverse reactions is highly variable, ranging from 3% to 88%, depending on factors like type of antivenom used and differences in treatment protocols and patients’ characteristics [129]. A study by Stone et al. showed that antivenom reactions are possibly due to IgG immunoglobulin complexes and impurities in the antivenom, so the manufacturing seems to play a pivotal role in the development of early adverse reactions [130]. Furthermore, the administration of a small dose of antivenom to identify patients who may develop early adverse drug reactions (ADR) has been proven to not constitute a sensitive or specific test, and can also itself cause anaphylactic reactions [131].

To prevent severe early reactions and anaphylaxis, premedication is commonly used. It generally consists of the early administration of hydrocortisone and antihistamines before antivenom infusion. Although it is a very diffused practice, there is no clear evidence of its benefits, and only adrenaline has proved to be safe and effective in preventing severe reactions [132]. Moreover, the EEACI guidelines clearly suggest that the subcutaneous injection of a low dose of adrenaline can prevent anaphylaxis when snake antivenom is administered [106].

Among the three most administered antivenoms in Europe—European Viper Venom Antiserum*^®^* (discontinued), ViperFAV*^®^*, and ViperaTAb*^®^*)—ViperaTAb*^®^* was associated with the lowest incidence of adverse reactions, thanks to the combination of low protein load and short half-life [121]. However, studies on antivenoms’ safety profiles are predominantly retrospective and performed remotely, increasing the risk of underreporting of adverse reactions.

Although no specific contraindications for antivenom administration are available, patients with a history of reactions to equine or ovine derivatives, and/or with a history of atopy (e.g., atopic dermatitis or asthma), are at a higher risk of developing anaphylaxis. In the case of acute anaphylaxis during antivenom administration, this must be suspended immediately, and the patient should be treated with a specific protocol, such as the one typically applied for anaphylactic shock arising from other etiologies (see Figure 1 in Muraro et al. [106]).

The WHO defines as ”late reactions” as those occurring between 5 and 24 days after antivenom administration. They correspond to a syndrome called “serum sickness” (type III hypersensitivity in the Gell and Coombs classification), where the immune system of the patient mounts an IgG- or IgM-based antibody response towards the heterologous proteins of the antivenom [23]. The antivenom protein concentration and dosage plays a key role in the development of this reaction, which is more common for IgG antivenoms and F(ab’)_2_ antivenoms. The most common clinical manifestations of this syndrome are fever, myalgia, arthralgia, arthritis, urticaria, lymphadenopathy, and gastrointestinal disorders. Additionally, laboratory tests show elevated erythrocyte sedimentation rate, leukocytosis occasionally accompanied by eosinophilia, hematuria, proteinuria, and decrease in complement components in serum (e.g., C3, C4, CH50 activity) [128]. In clinical practice, when the patient who had been administered snake antivenom during his hospital stay is discharged, an evaluation by the general practitioner is recommended if the previous mentioned symptoms appear within one month, in order to perform a blood test and start an adequate treatment if serum sickness is suspected. A telephonic follow-up is usually performed by clinical toxicologists. 

Antivenoms should always be available in major hospitals or in peripheral hospitals of areas considered at risk for viper bite. In 2014, the Italian Pharmaceutical Agency (AIFA) authorised the importation of viper antivenoms from foreign countries, due to their unavailability in Italy [133]. Although every national PCC has its own antivenom stockpile, that can be mobilised in case of necessity to the nearest hospital, every hospital can provide its own antivenom after an official authorisation from the National Pharmaceutical Agency. It is always advisable that clinicians who are managing a patient with a suspected viper bite ask for the specialistic consultation of a toxicologist to discuss the treatment and the possible administration of antivenom when available. 

**Figure 3:**
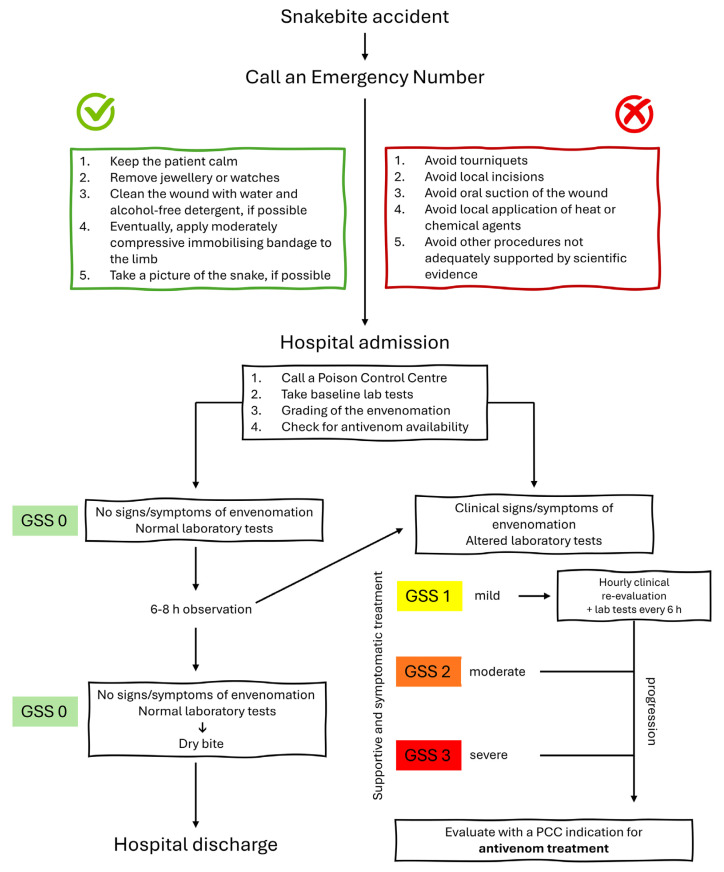
Simplified flowchart for the clinical management of *Vipera* snakebite. PCC = Poison Control Centre; GSS = Grading Severity Score.

Below is a simplified flowchart for managing patients with suspected snakebites. It is important to remember that if antivenom administration is necessary, consulting the Poison Control Centre for individual dosage guidance is crucial (Figure 3).

## 3. Discussion

Snakes belonging to the *Vipera* genus are frequently implicated in snakebite accidents in Europe (e.g., [11,13,28]). In Italy, four species of venomous snakes from this genus are present, all officially recognised as medically relevant by the WHO: *V. ammodytes*, *V. aspis*, *V. berus*, and *V. ursinii* [35,75,76,77]. While envenomations by these species are uncommon and rarely reported, their impact is likely underestimated due to factors such as the lack of mandatory reporting of snakebite incidents in Europe and limited physician training in identifying snakebites and administering appropriate treatment [134]. In fact, although rare, bites by the *Vipera* species present in Italy can potentially lead to serious consequences and require prompt hospitalisation and specialised toxicological assessment (e.g., [17,19,98,107,135,136]).

While a protocol for the management and treatment of viper bites at the European level has been proposed [14], differences in venomous snake species and available resources, including antivenoms, in each country highlight the need for nation-specific protocols to optimise snakebite treatment locally. In this scenario, Italian clinicians need tools to aid in the management of *Vipera* envenomations. In the present work, we thus report a detailed, country-specific update to the protocol for the clinical management of snakebite in Italy, aiming to standardise and simplify medical procedures in cases of viper envenomation in the country.

We provide a taxonomic key to facilitate quick and reliable identification of the snake species causing the envenomation, which can help with predicting ensuing symptoms and facilitate decision making when administering antivenom treatment [10,137,138]. Additionally, we offer an overview of the venom components of *V. ammodytes*, *V. aspis*, *V. berus*, and *V. ursinii*, potentially helpful in predicting clinical outcomes. Major toxin families such as SVMP, PLA_2_, SVSP, and CTL account for most of the compositions of the venoms of these species [27,43,51,61,70]. In line with this, Italian vipers primarily induce haemorrhagic and cytotoxic effects (typical of viper envenomation [10,20]), although neurotoxic symptoms can also occur in some cases [40,54]. In fact, clinical manifestations range from local symptoms (e.g., algesia, swelling) requiring topical treatment to systemic, potentially lethal complications (e.g., renal failure, haematological alterations) necessitating antivenom therapy [11,13,77].

In light of this, we present a tool for Italian healthcare providers to support them when applying treatment, including an overview of first aid procedures on the field and guidelines for the administration of antivenom. Nonetheless, we stress that despite the presence of effective, generally well-tolerated antivenoms, the management of snakebites remains a challenging issue [11], and caution should always be exercised when contemplating antivenom administration due to the potential for severe side effects [128]. Although the protocol presented herein was designed to specifically treat snakebite in Italy, the methodology described can likely be extended to other European states, considering local drug and antivenom availability. Nonetheless, adaptation to local resources is essential.

In order to improve the efficacy of the present protocol and, in general, the management of emergency situations associated with viper bites in Italy, enhancing our understanding of the epidemiology of snakebites in the country is crucial. We advocate the need for Italian clinicians to report snakebite cases to establish comprehensive statistics on viper bites and associated clinical features. To this end, the creation of a unified database to collect statistics on snakebites in the country, currently missing, would surely be advantageous. Finally, collaboration among the various PCCs existing in the national territory should be encouraged to optimise the application of correct management protocols and improve the accuracy of statistics in this field.

## 4. Materials and Methods

To acquire insights into the management of *Vipera* snakebites in Italy, a comprehensive literature review was conducted from December 2023 to April 2024. PubMed, Scopus, and Google Scholar databases were systematically searched using the following query: “Vipera” AND “snakebite” AND “treatment” AND (“Italy” OR “Europe”). Guidelines, textbooks, and peer-reviewed articles were carefully examined for relevant information. The data extracted from these sources were analysed and discussed among the authors, who possess expertise in herpetology, toxicology, and medicine. Additionally, insights into the practical clinical management of snakebites in Italy were obtained from co-authors who are experienced physicians. Subsequently, all gathered information underwent thorough scrutiny for consistency and relevance to Italy’s medical infrastructure. The data were rigorously discussed, taking into account the current medical resources available in the country. Finally, the refined information was synthesised and summarised to offer an optimised update of *Vipera* snakebite management in Italy. 

## Figures and Tables

**Table 1:** Italian Poison Control Centres and Contact Information. Available at: https://www.iss.it/sostanze-chimiche-tutela-salute/-/asset_publisher/LDhvJczHbcsw/content/centri-antiveleni-e-intossicazioni-da-sostanze-chimiche accessed on 20 May 2024.

City	Hospital	Telephone Number
Bergamo	Azienda Ospedaliera Papa Giovanni XXIII	800.88.33.00
Firenze	Azienda Ospedaliera Careggi	(+39) 055.794.7819
Foggia	Azienda Ospedaliera Universitaria di Foggia	800.183.459
Milano	Ospedale Niguarda	(+39) 02.66.1010.29
Napoli	Azienda Ospedaliera A. Cardarelli	(+39) 081.545.3333
Pavia	Istituti Clinici Scientifici Maugeri	(+39) 0382.24.444
Roma	Ospedale Pediatrico Bambino Gesù	(+39) 06.6859.3726
	Policlinico Umberto I	(+39) 06.4997.8000
	Policlinico A. Gemelli	(+39) 06.305.4343
Verona	Azienda Ospedaliera Universitaria Integrata Verona	800.011.858

**Table 2:** Comparison between French and Swedish envenomation grading systems.

Grade	French Grading System (GSS)	Swedish Grading System (Stockholm Criteria)
**0—No envenoming**	Fang marks with no oedema, no local reaction.	
1—Minimal */mild **	Local oedema around the bite site with no systemic symptoms or signs.	* Local swelling without systemic effects.** Local or more extensive oedema with or without gastrointestinal signs and symptoms but without other systemic effects.
**2—Moderate**	Regional oedema and or haematoma. Moderate systemic symptoms/signs: Mild hypotension, vomiting, diarrhoea, neurotoxicity.	Extensive oedema. Shock lasting less than 2 h, other systemic features such as transient cardiac arrhythmia, neurotoxicity, pronounced gastrointestinal symptoms.
**3—Severe**	Extensive oedema involving the trunk. Severe systemic symptoms/signs: prolonged hypotension, shock, bleeding complications, organ failure.	Oedema involving the trunk. Life-threatening systemic symptoms and signs. Shock lasting longer than 2 h.
**4**		Death

## Data Availability

No new data were created or analysed in this study. Data sharing is not applicable to this article.

## Supplementary Materials

The following supporting information can be downloaded at: https://www.mdpi.com/article/10.3390/toxins16060255/s1, Table S1: List of species and subspecies of Italian snakes. Based on Di Nicola et al. (2021), Sindaco and Razzetti (2021) and Fritz and Ihlow (2022) [35,77,139]; Table S2: Characteristics of the six antivenoms currently available in Italy, using information provided in the safety data sheets; Figure S1: Photos of the species and subspecies of Italian non-viperid snakes: *Indotyphlops braminus* (A) and *Eryx jaculus* (B). For each taxon, examples of dorsal pattern, head portrait, and dorsal scales are shown. Modified from Di Nicola (2019) [76]; Figure S2: Photos of the species and subspecies of Italian non-viperid snakes: *Malpolon insignitus insignitus* (A), *M. monspessulanus monspessulanus* (B), *Natrix helvetica cetti* (C), *N. h. sicula* (D), *N. maura* (E), *N. natrix vulgaris* (F), *N. tessellata* (G). For each taxon, examples of dorsal pattern, head portrait, and dorsal scales are shown. Modified from Di Nicola (2019) [76]; Figure S3: Photos of the species and subspecies of Italian non-viperid snakes: *Coronella austriaca austriaca* (A), *C. girondica* (B), *Elaphe quatuorlineata quatuorlineata* (C), *Hemorrhois hippocrepis* (D), *Hierophis viridiflavus viridiflavus* (E), *H. v. carbonarius* (F), *Macroprotodon* cf. *cucullatus* (G). For each taxon, examples of dorsal pattern, head portrait, and dorsal scales are shown. Modified from Di Nicola (2019) [76]; Figure S4: Photos of the species and subspecies of Italian non-viperid snakes: *Telescopus fallax fallax* (A), *Zamenis lineatus* (B), *Z. longissimus* (C), *Z. situla* (D). For each taxon, examples of dorsal pattern, head portrait, and dorsal scales are shown. Modified from Di Nicola (2019) [76]; Figure S5: Mid-body dorsal scales count on *Vipera ammodytes* shed skin. Image from Di Nicola (2019) [76].; Figure S6: Nomenclature of the scales on the belly and under the tail in *Vipera berus*. Image from Di Nicola (2019) [76]; Figure S7: Nomenclature of the main head scales in a viper (*Vipera berus*): dorsal (A); lateral (B); ventral (C). First ventral scale according to Dowling (1951). Image from Di Nicola (2019) [76,140].
