# Supplementary material for: A Guide to the Clinical Management of Vipera Snakebite in Italy
Source: Toxins (Basel). 2024 May 31;16(6):255. doi: 10.3390/toxins16060255 (PMC11209566; doi:10.3390/toxins16060255)
Supplement: Supplementary file 1 [file toxins-16-00255-s001.zip › toxins-3014738-supplementary.pdf]

A guide to the Clinical Management of *Vipera* Snakebite in Italy

SUPPLEMENTARY MATERIALS

Table S1: List of species and subspecies of Italian snakes. Based on Di Nicola et al. (2021) [77], Sindaco & Razzetti (2021) [35] and Fritz & Ihlow (2022) [139].

**Family**	**Species**	**Subspecies**	**Figure**
Typhlopidae Merrem 1820	*Indotyphlops braminus* (Daudin 1803)	*Indotyphlops braminus* (Daudin 1803)	S1 A
Erycidae Bonaparte 1840	*Eryx jaculus* (Linnaeus 1758)	*Eryx jaculus* (Linnaeus 1758) subspecific framework to be defined	S1 B
Psammophiidae Boie 1827	*Malpolon insignitus* (Geoffroy Saint-Hilaire 1827)	*Malpolon insignitus insignitus* (Geoffroy Saint-Hilaire 1827)	S2 A
	Malpolon monspessulanus (Hermann 1804)	*Malpolon monspessulanus monspessulanus* (Hermann 1804)	S2 B
Natricidae Bonaparte 1840	*Natrix helvetica* (Lacépède 1789)	*Natrix helvetica cetti* Gené 1839	S2 C
		Natrix helvetica sicula (Cuvier 1829)	S2 D
	Natrix maura (Linnaeus 1758)	*Natrix maura* (Linnaeus 1758) monotypic	S2 E
	Natrix natrix (Linnaeus 1758)	*Natrix natrix vulgaris* Laurenti 1768	S2 F
	Natrix tessellata (Laurenti 1768)	*Natrix tessellata* (Laurenti 1768) monotypic	S2 G
Colubridae Oppel 1811	*Coronella austriaca* Laurenti 1768	*Coronella austriaca austriaca* Laurenti 1768	S3 A
	Coronella girondica (Daudin 1803)	*Coronella girondica* (Daudin 1803) monotypic	S3 B
	Elaphe quatuorlineata (Bonnaterre 1790)	*Elaphe quatuorlineata quatuorlineata* (Bonnaterre 1790)	S3 C
	Hemorrhois hippocrepis (Linnaeus 1758)	*Hemorrhois hippocrepis* (Linnaeus 1758) monotypic	S3 D
	Hierophis viridiflavus (Lacépède 1789)	*Hierophis viridiflavus viridiflavus* (Lacépède 1789)	S3 E
		Hierophis viridiflavus carbonarius (Bonaparte 1833)	S3 F
	Macroprotodon cf. *cucullatus* (Geoffroy Saint-Hilaire 1827)	*Macroprotodon* cf. *cucullatus* (Geoffroy Saint-Hilaire 1827)	S3 G
	Telescopus fallax (Fleischmann 1831)	*Telescopus fallax fallax* (Fleischmann 1831)	S4 A
	Zamenis lineatus (Camerano 1891)	*Zamenis lineatus* (Camerano 1891) monotypic	S4 B
	Zamenis longissimus (Laurenti 1768)	*Zamenis longissimus* (Laurenti 1768) monotypic	S4 C
	Zamenis situla (Linnaeus 1758)	*Zamenis situla* (Linnaeus 1758) monotypic	S4 D
Viperidae Oppel 1811	*Vipera ammodytes* (Linnaeus 1758)	*Vipera ammodytes ammodytes* (Linnaeus 1758)	2 A
	Vipera aspis (Linnaeus 1758)	*Vipera aspis aspis* (Linnaeus 1758)	2 B
		Vipera aspis francisciredi (Laurenti 1768)	2 C
		Vipera aspis hugyi (Schinz 1834)	2 D
	Vipera berus (Linnaeus 1758)	*Vipera berus marasso* (Pollini 1818)	2 E
		Vipera berus walser Ghielmi, Menegon, Marsden, Laddaga & Ursenbacher 2016	2 F
	Vipera ursinii (Bonaparte 1835)	*Vipera ursinii ursinii* (Bonaparte 1835)	2 G

Table S2: Characteristics of the six antivenoms currently available in Italy. Information obtained from the corresponding safety data sheets.

Antivenom name	Marketing Authorisation Holder (MAH)	Source	Type	Vipera spp raised against	Recognised by WHO[§]	Antibody concentration	Affinity purified	Formulation	Recommended dose and route of administration by MAH	Total amount of protein per dose	Storage instructions	Shelf-life of the antivenom
**Viper Venom Antitoxin®**	Biomed Sera and Vaccines Manufacturing Company, Warsaw, Poland	Equine	$F(ab')_2$	*V. berus*	Yes	NR	No	Injectable solution, ampoule of 5 ml	5 mL IM	450-850 mg	Refrigerator (+2°C ÷ +8°C)	36 months
**ViperaTAb®**	MicroPharm Ltd UK	Ovine	Fab	*V. berus*	No	25 mg/mL	Yes	Injectable solution, bottle of 100 mg/4 mL	8 mL IV	200 mg	Refrigerator (+2°C ÷ +8°C)	24 months
**ViperFAV®**	Sanofi Pasteur, France	Equine	$F(ab')_2$	*V. berus, V. aspis, V. ammodytes*	Yes	99-116 mg/mL	No	Injectable solution, bottle of 4 mL	4 mL IV	396-468 mg	Refrigerator (+2°C ÷ +8°C)	36 months
**Snake Venom Antiserum®**	Bul Bio NCIPD Ltd, Sofia, Bulgaria	Equine	$F(ab')_2$	*V. ammodytes*	No	NR	No	Injectable solution, bottle 100 AU	10 mL SC and 10 mL IM	900-1700 mg	Refrigerator (+2°C ÷ +8°C)	24 months
**Viekvin®**	Institute of Virology, Vaccines and Sera "Torlak", Belgrade, Serbia	Equine	$F(ab')_2$	*V. ammodytes*	Yes	NR	No	Injectable solution, ampoule of 5 mL	5-10 mL IM	NR	Refrigerator (+2°C ÷ +8°C)	36 months
**Vetal Polisera®***	Vetal Serum and Biological Products Manufacturig Industry and Commerce, Andiyaman, Turkey	Equine	$F(ab')_2$	*V. ammodytes, Macrovipera lebetina, Montivipera xanthina*	No	NR	No	Injectable solution, ampoule of 10 mL	10-50 mL IM or IV in severe envenoming	NR	Refrigerator (+2°C ÷ +8°C)	24 months

IM: intramuscular; IV: intravenous; NK: not known; NR: not reported; SC: subcutaneous; WHO: World Health Organization. *No usage data available for Italy. [§]WHO, 2016. Venomous snakes distribution and species risk categories. Geneva.

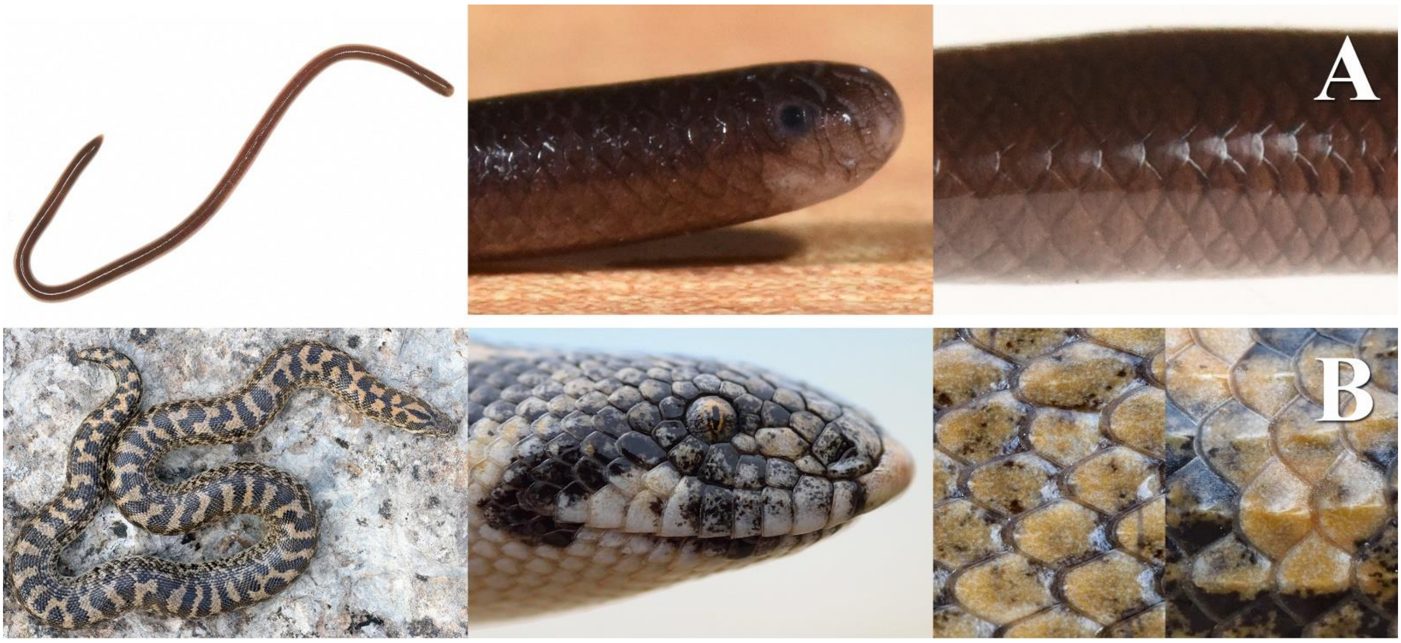

Figure S1: Photos of the species and subspecies of Italian non-viperid snakes: *Indotyphlops braminus* (A) and *Eryx jaculus* (B). For each taxon, examples of dorsal pattern, head portrait, and dorsal scales are shown. Modified from Di Nicola (2019) [76].

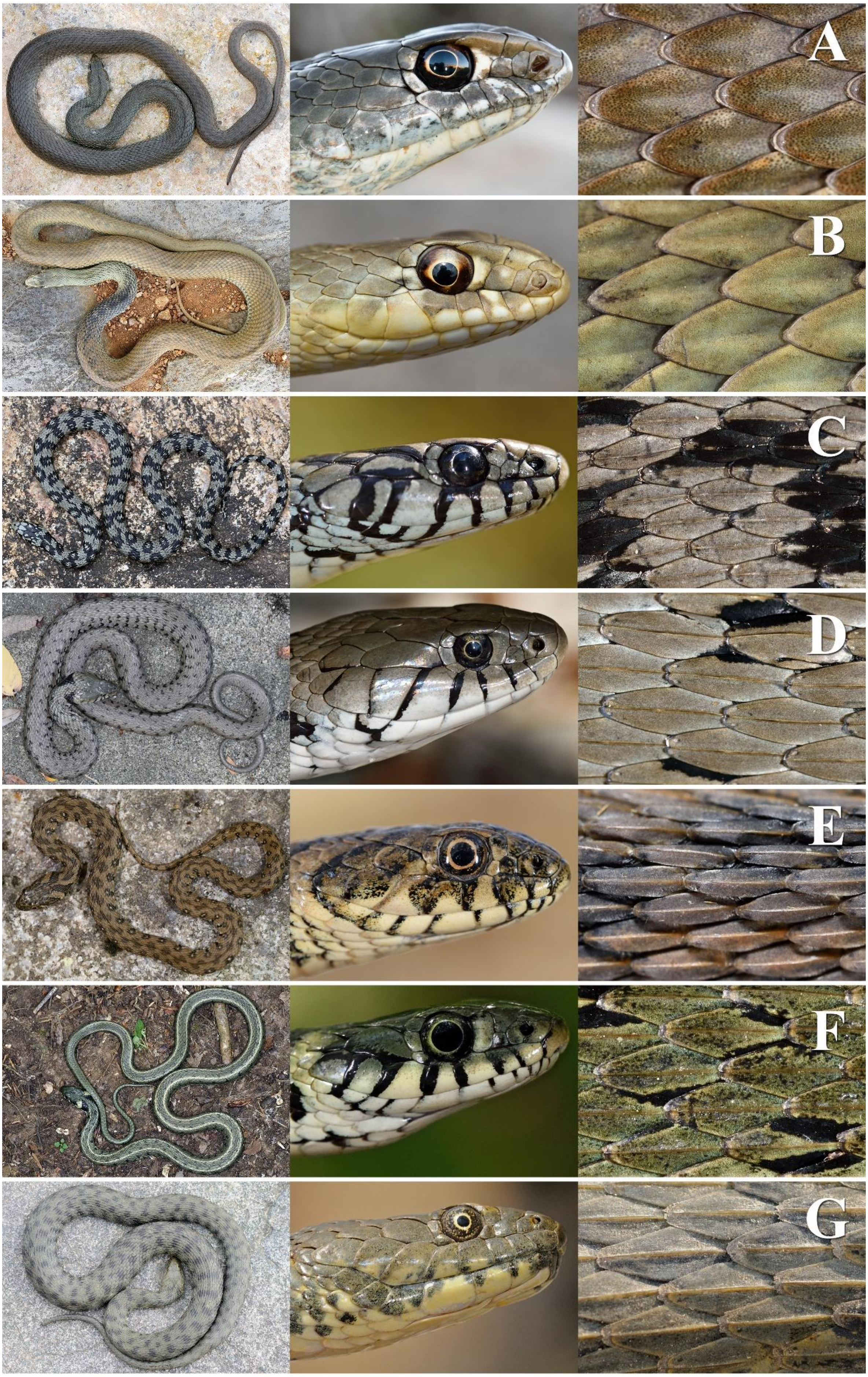

Figure S2: Photos of the species and subspecies of Italian non-viperid snakes: *Malpolon insignitus insignitus* (A), *M. monspessulanus monspessulanus* (B), *Natrix helvetica cetti* (C), *N. h. sicula* (D), *N. maura* (E), *N. natrix vulgaris* (F), *N. tessellata* (G). For each taxon, examples of dorsal pattern, head portrait, and dorsal scales are shown. Modified from Di Nicola (2019) [76].

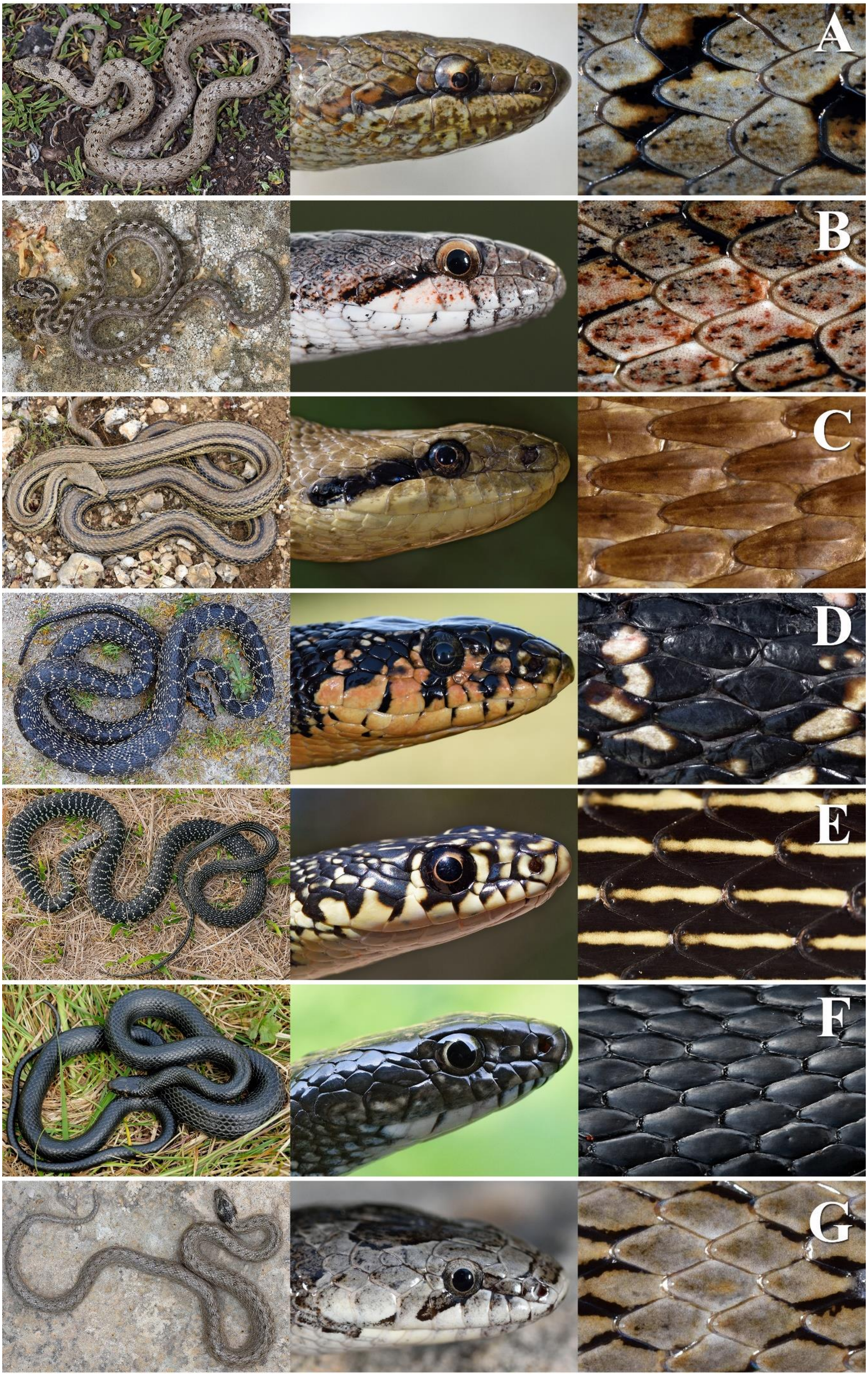

Figure S3: Photos of the species and subspecies of Italian non-viperid snakes: *Coronella austriaca austriaca* (A), *C. girondica* (B), *Elaphe quatuorlineata quatuorlineata* (C), *Hemorrhois hippocrepis* (D), *Hierophis viridiflavus viridiflavus* (E), *H. v. carbonarius* (F), *Macroprotodon* cf. *cucullatus* (G). For each taxon, examples of dorsal pattern, head portrait, and dorsal scales are shown. Modified from Di Nicola (2019) [76].

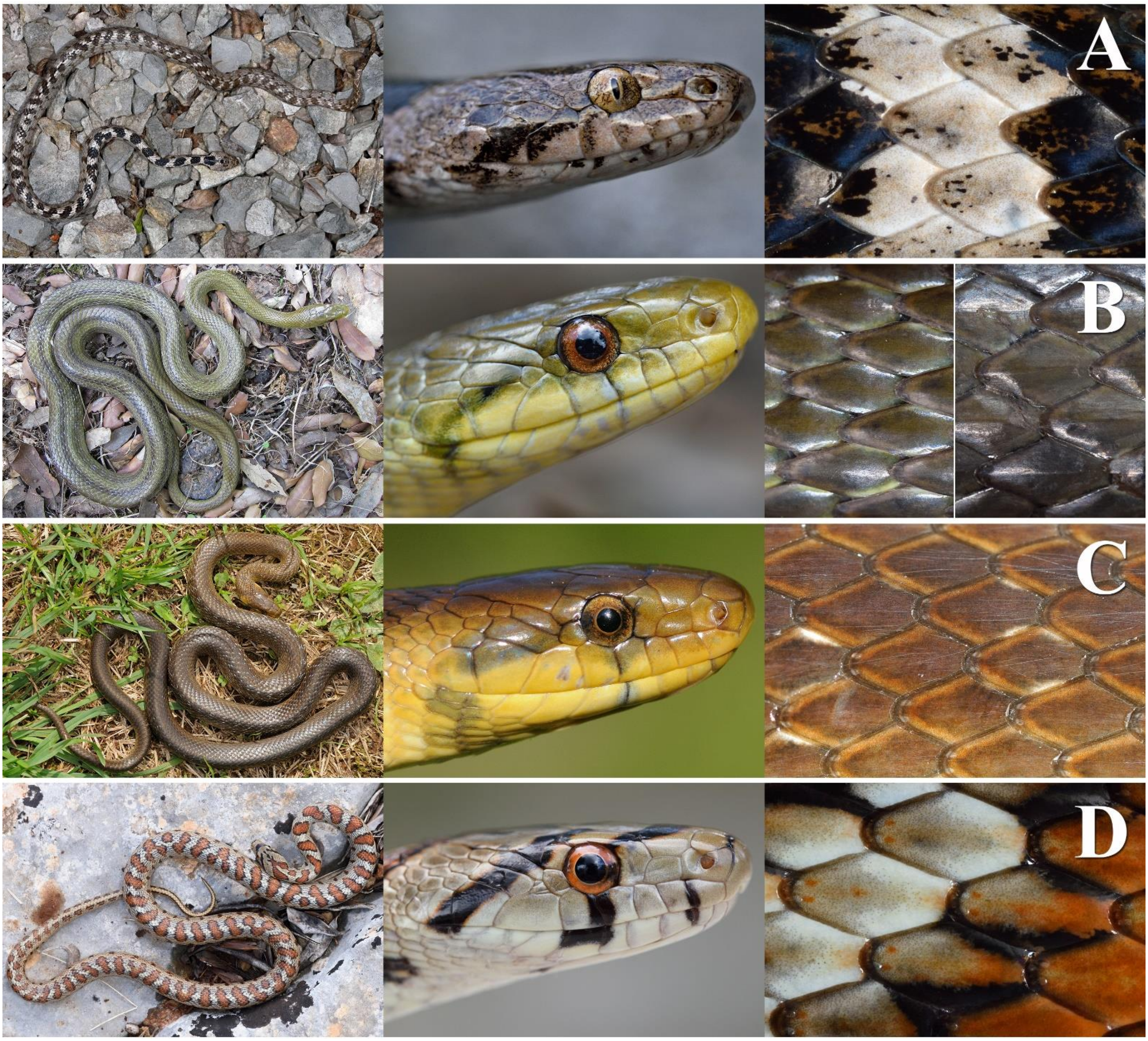

Figure S4: Photos of the species and subspecies of Italian non-viperid snakes: *Telescopus fallax fallax* (A), *Zamenis lineatus* (B), *Z. longissimus* (C), *Z. situla* (D). For each taxon, examples of dorsal pattern, head portrait, and dorsal scales are shown. Modified from Di Nicola (2019) [76].

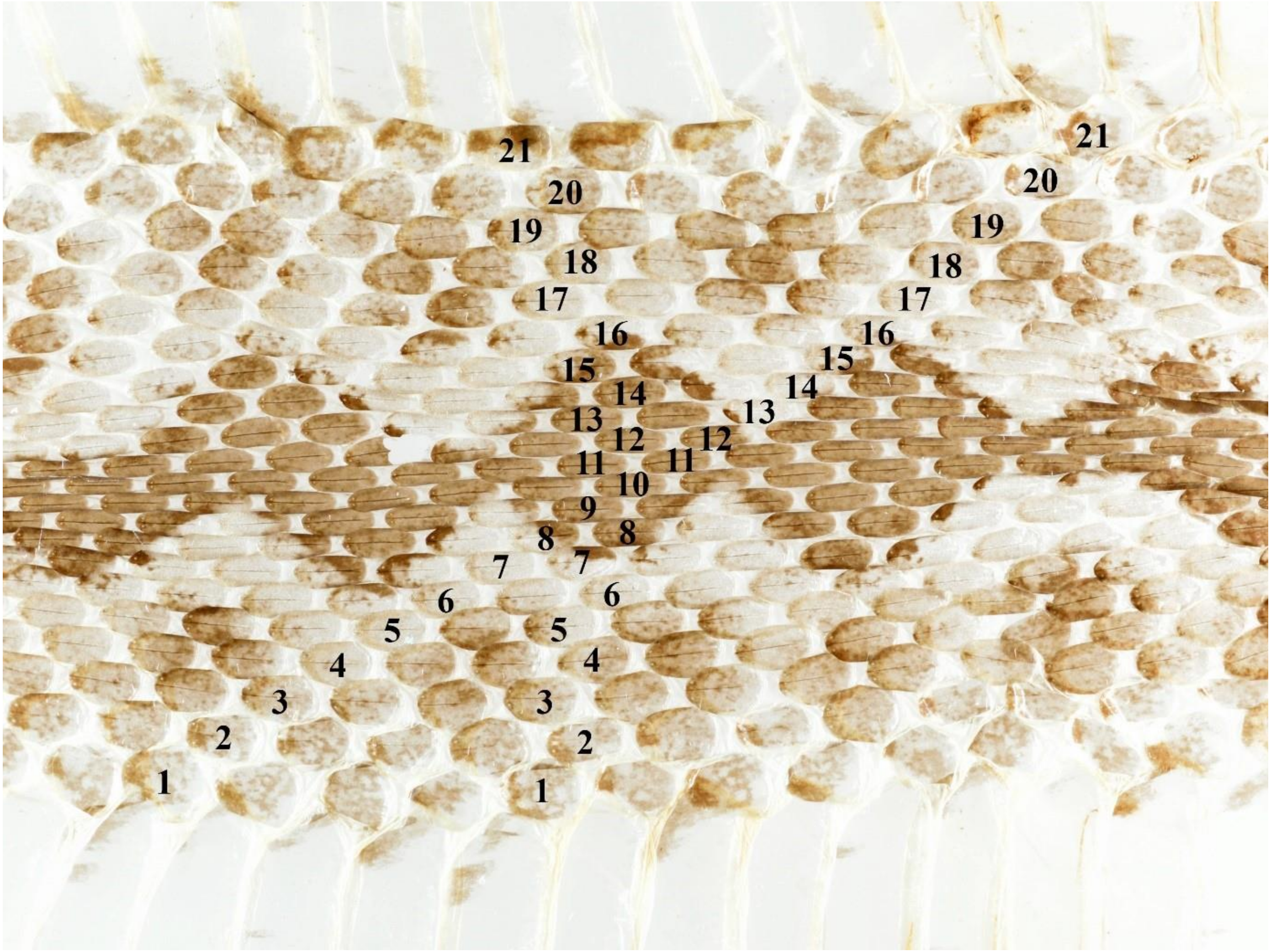

Figure S5: Mid-body dorsal scales count on *Vipera ammodytes* shed skin. Image from Di Nicola (2019) [76].

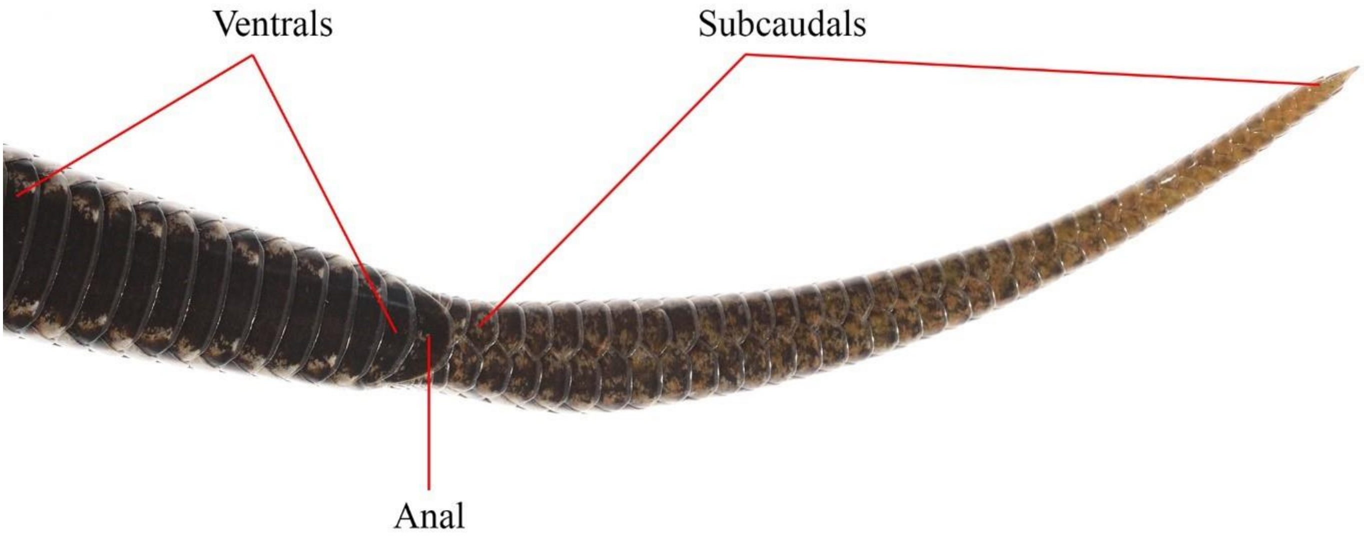

Figure S6: Nomenclature of the scales on the belly and under the tail in *Vipera berus*. Image from Di Nicola (2019) [76].

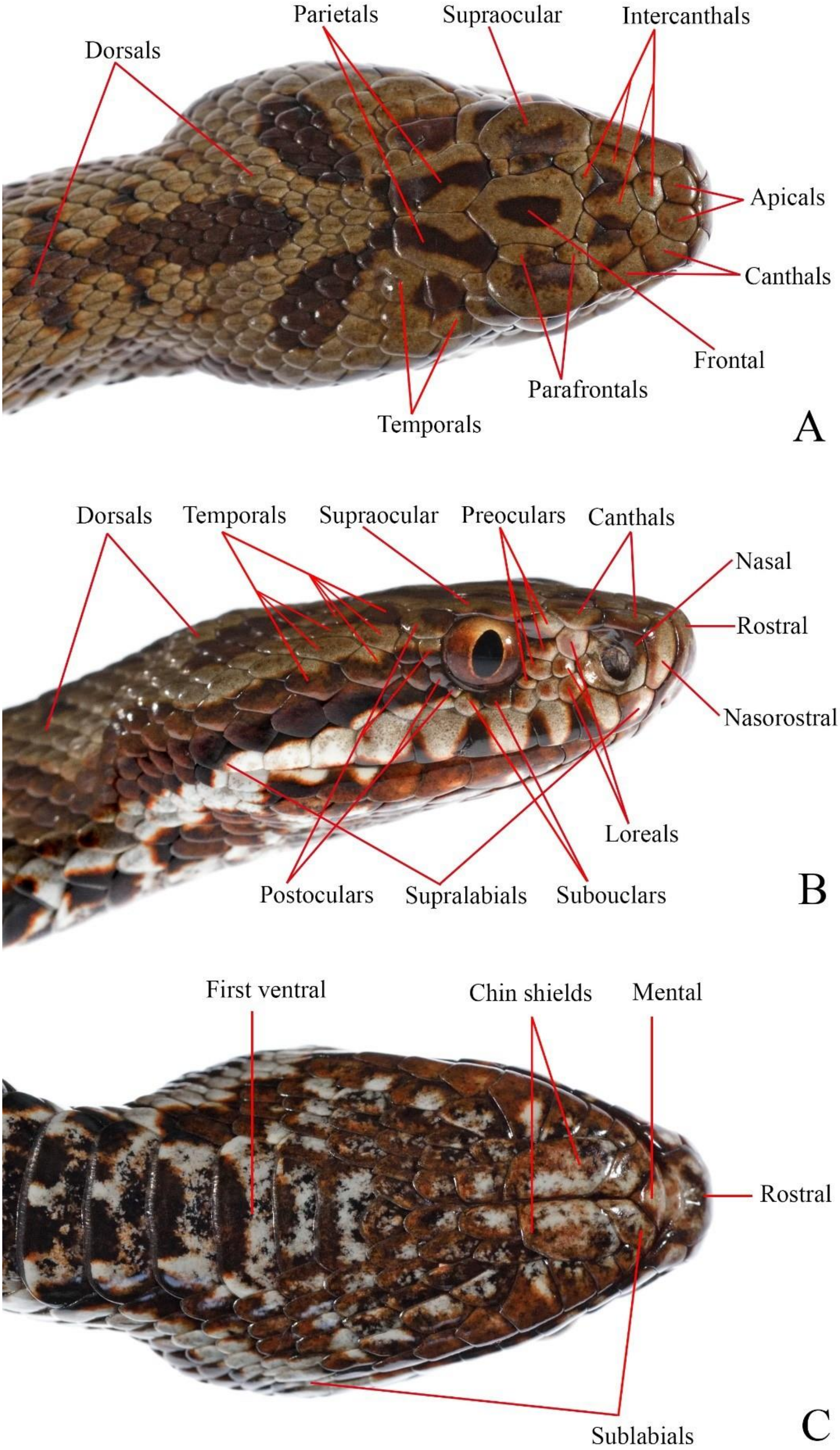

Figure S7: Nomenclature of the main head scales in a viper (*Vipera berus*): dorsal (A); lateral (B); ventral (C). First ventral scale according to Dowling (1951) [140]. Image from Di Nicola (2019) [76].
